# Liquid Metal Based Flexible and Implantable Biosensors

**DOI:** 10.3390/bios10110170

**Published:** 2020-11-10

**Authors:** Mingkuan Zhang, Xiaohong Wang, Zhiping Huang, Wei Rao

**Affiliations:** 1 Chinese Academy of Sciences Key Laboratory of Cryogenics, Technical Institute of Physics and Chemistry, Beijing 100190, China; zhangmingkuan17@mails.ucas.ac.cn (M.Z.); wangxiaohong19@mails.ucas.ac.cn (X.W.); 2 Beijing Key Lab of CryoBiomedical Engineering and Key Lab of Cryogenics, Beijing 100190, China; 3 School of Engineering Science, University of Chinese Academy of Sciences, Beijing 100039, China; 4 School of Future Technology, University of Chinese Academy of Sciences, Beijing 100049, China; 5 Department of Mechanical Engineering, Imperial College London, London SW7 2BU, UK; zhiping.huang16@imperial.ac.uk

**Keywords:** liquid metal, biosensor, flexible, implantable, mechanical, gas, temperature, electrical, optical

## Abstract

Biosensors are the core elements for obtaining significant physiological information from living organisms. To better sense life information, flexible biosensors and implantable sensors that are highly compatible with organisms are favored by researchers. Moreover, materials for preparing a new generation of flexible sensors have also received attention. Liquid metal is a liquid-state metallic material with a low melting point at or around room temperature. Owing to its high electrical conductivity, low toxicity, and superior fluidity, liquid metal is emerging as a highly desirable candidate in biosensors. This paper is dedicated to reviewing state-of-the-art applications in biosensors that are expounded from seven aspects, including pressure sensor, strain sensor, gas sensor, temperature sensor, electrical sensor, optical sensor, and multifunctional sensor, respectively. The fundamental scientific and technological challenges lying behind these recommendations are outlined. Finally, the perspective of liquid metal-based biosensors is present, which stimulates the upcoming design of biosensors.

## 1. Introduction

The biosensor is a typically integrated unit that senses specific bio-sensitive substances and converts them into a measurable signal for detection. There are several parts included: bio-sensitive materials as a biorecognition element, a physical or chemical transducer element, a signal transmission, and an amplifier element. The signal input could be biophysical (light [1], sound [2], temperature [3], force [4], smell [5], and so on) or biochemical (small molecules like DNA [6], enzymes [7], cells [8], and macromolecules like sweat, blood, and even tears [9]). According to the type of transducer, it can be classified as an electrode, fluorescence, field-effect transistor, or surface plasma resonance [10]. The advantages of biosensors such as good selectivity, high sensitivity, fast analysis speed, low cost, high miniaturization and integration, enable them to be widely applied in the biomedical, food industry, environmental monitoring, and other fields.

Traditional biosensors serve as common auxiliary or adjunctive sensing equipment of the biological body. However, a higher demand for dynamic and real-time sensing propels the development of soft biosensor. Flexible electronic technologies have been demonstrated as cost-effective approaches for realizing the large-scale production of biosensors integration. Hence, the soft biosensor is promising and has gained considerable attention recently based on the rapid development of material technology and flexible electronics.

Selecting suitable materials according to different signals is the key to sensitive and precise enhancement. For example, nanomaterial-based biosensors have been developed and widely investigated in biology. Various types of nanoparticles are embedded in the biosensor with different functions as a carrier [11], catalyst [12], signal amplification [13], and signaling molecule [14]. Besides, numerous soft materials are explored for soft or flexible biosensors, such as polyimide (PI) and polyethylene terephthalate (PET) [15], polydimethylsiloxane (PDMS) [16], hydrogels [17], carbon-based materials including graphene [18], and carbon nanotubes [19] They serve as a mechanical support for integrating electronic circuits, playing an important role in biosensors.

For biosensors, especially soft biosensors, it is vitally important to perform flexible properties like mechanical stability and biocompatibility, which demands better performance from materials [20]. Liquid metal (LM) and its micro/nano particles are investigated as newly emerged functional materials owning to their unique features that are superior to other materials and additional functions that conventional micro/nano material fail to achieve. For example, their self-healing quality endows liquid metal micro/nano-based composites with stronger durability and robustness which are essential for implantable sensors. Besides, Ga nanoparticles have been proven to have good biocompatibility which is necessary for implantable sensors. A relatively high sensitivity may be obtained from the nanomaterial-based biosensors attributed to their larger surface area and effective contact area. However, the limitation of reproductivity and stability restricts their wide application [15]. Meanwhile, for bulk liquid metal, the fabrication process is much more convenient. The electrical properties and mechanical behavior of bulk liquid metal are also more stable and superior. The low Young’s modulus ensures that small forces are required to achieve large deformation. As a type of metal with a low melting point, liquid metal shows higher electrical conductivity than most soft polymer materials [21]. At the same time, bulk liquid metal can achieve stable electrical signal transmission under deformation of ~700%. The resistive and capacitive sensors based on liquid metal have been designed for temperature [3], gas [22], and force [23] sensing. Moreover, the intrinsic flexibility of liquid metal provides biosensors with additional functionality in wearable electrical sensors. Liquid metal-based soft circuits can maintain mechanical stability under stretching or bending in health-monitoring and motion-sensing applications [24]. The on-skin biosensors conformally contact with the body surface and realize dynamical sensing. Besides, it is possible to utilize liquid metal in implantable sensors due to its inherent biocompatibility, as soft sensors applied in biomedicine require biomaterial with nontoxicity and conformality, for example, artificial muscle, electrical skin, and nerve connection. It is also noteworthy that fabricating liquid metal soft circuits on different substrates through printing or direct writing is much easier than the traditional solid circuit. Overall, liquid metal is a favorable candidate as a transducing-elements in biosensors, especially as soft electrodes and electrical interconnection.

This article intends to introduce the biosensors based on liquid metal, providing a comprehensive overview of characteristics and material fabrication. Recent progress on the liquid metal biosensor is illustrated as well. Finally, existing challenges and future perspectives are discussed for further investigation.

## 2. Properties

Liquid metal has played an important role in developing bio-sensors due to their unique physical and chemical properties. Therefore, the properties of liquid metal related to biosensing will be introduced in the following chapters. The liquid metal in this article mainly refers to gallium (Ga), indium (In), tin (Sn), and their alloys.

### 2.1. Morphology

**Figure 1:**
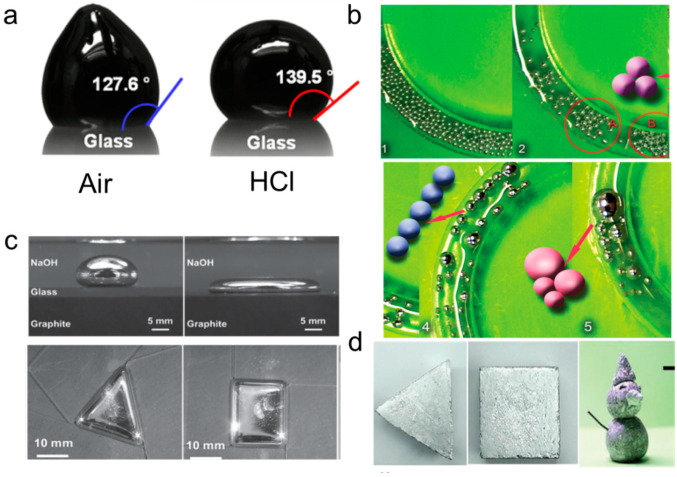
Liquid metal morphology and its control methods. (**a**) The morphology of liquid metal droplets with and without oxide. Reprinted with permission from [26]. Copyright (2013) American Chemical Society. (**b**) The dispersion and merge of liquid metal [27]. Red circle A and B shows the liquid metal droplets about to reunite. Copyright (2015) WILEY-VCH. (**c**) The transformation of liquid metal droplets on graphite with a different shape in an electrolyte [30]. Copyright (2015) WILEY-VCH. (**d**) Different shapes of oxide-rich liquid metal [36]. Copyright (2020) WILEY-VCH.

The surface tension of Ga-based LM could reach ~600 mN/m, which is 9 times larger than that of water. Meanwhile, the Young’s modulus of liquid metal is as low as 1–10 Pa which is 10^7^ times less than that of steel (at 20 °C) [25]. According to the mechanism of decreasing surface energy, bulk liquid metal without oxide trends to be a sphere, and liquid metal droplets are inclined to merge (Figure 1a) [26]. Meanwhile, bulk liquid metal at the liquid state is easily broken into mini or microdroplets when force is applied (Figure 1b) [27]. Clearly, it is hard to regulate the shape of liquid metal because of its low Young’s modulus, low melting point, and high surface tension. Although liquid metal demonstrates the advantages of flexibility and deformability, low adhesion makes it easy to be removed from the substrates [28,29].

Reducing the surface tension of liquid metal by generating oxide film can regulate its morphology. Applying an electric field or adding a strong oxidant could accelerate the oxidation of the liquid metal and realize the adjustment of its surface morphology (Figure 1c) [30,31,32]. However, the solution environment and the applied external electric field make this control method incompatible with biosensors. Another way is mixing liquid metal with micro/nanoparticles by using a mechanical stirring method or electrochemical method [33,34]. Particles embedded in liquid metal could intensely alter its properties. Higher dynamic viscosity, higher adhesion, and lower surface tension can be obtained while conductivity remains mostly consistent [35]. As a result, liquid metal can be easily molded into various shapes (Figure 1d) [36], and could be used to make sensor elements and sensor circuits [37]. In addition, many metal particles (Fe, Cu, and Ni) and liquid metal may form an intermetallic compound [28,38,39]. This may adjust the hardness and Young’s modulus of the mixture, and realize the self-solidification of the liquid metal.

### 2.2. Oxidation

**Figure 2:**
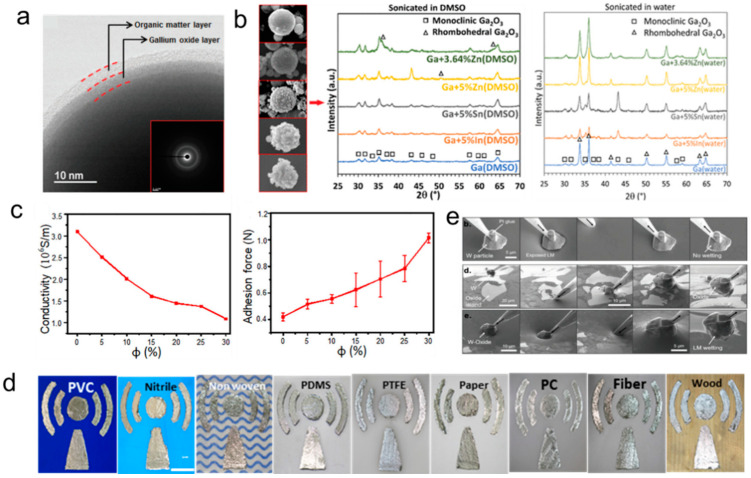
Oxidation of liquid metal. (**a**) TEM image to show the surface structure of GaInSn nanoparticles consisted of the gallium oxide layer and liquid metal core [40]. Copyright (2016) WILEY-VCH. (**b**) SEM images of liquid metal particles prepared by the ultrasonication method in DMSO. XRD patterns of different samples prepared in DMSO (the middle) and water (the right) [22]. Copyright (2016) American Chemical Society. (**c**) The conductivity of the mixture of Ni particles and EGaIn (the left) and the adhesion force (the left-right) [44]. Copyright (2018) WILEY-VCH. (**d**) The mixture of Ni particles and EGaIn was painted on various substrates [35]. Copyright (2018) Elsevier. (**e**) SEM to reveal the process of the oxide assisted wetting [45]. Copyright (2019) WILEY-VCH.

Ga can be readily oxidized when exposed to ambient air or a neutral aqueous solution. Liquid-state Ga and its alloys are homogenously covered by a thin native oxide layer, generally amorphous Ga_2_O_3_ (~3 nm) (Figure 2a) [40]. Different crystalline phases of gallium oxides generate in different environments after annealing. For example, monoclinic β-Ga_2_O_3_ crystal structure is easily formed after annealing when liquid metal was sonicated in DMSO, whereas liquid metal sonicated in DI water generates more particles covered with predominantly rhombohedral α-Ga_2_O_3_ (Figure 2b) [22]. β-Ga_2_O_3_ is a semiconductor material with a versatile wide bandgap (4.6 eV–4.9 eV) and has wide applications in wearable sensors and gas sensors [41]. Generally, the conductivity of β-Ga_2_O_3_ can be influenced by an ambient atmosphere. A high temperature activates the change of electronic consistency. At a low temperature, the oxygen-vacancies diffusion of β-Ga_2_O_3_ is frozen and the bulk electrical conductivity no longer responds to the change of circumstance gas composition [42].

Pure liquid metal has poor adhesion to most substrates, and only shows slight compatibility with polymer substrates, such as PVC and polydimethylsiloxane (PDMS) [43]. However, oxide endows liquid metal with superb adhesion with various substrates, and liquid metal can easily and steadily adhere to “oxide-phobic” substrates. Although a large amount of oxide in liquid metal will reduce the electrical conductivity and thermal conductivity, using oxide-rich liquid metal can enable the easy fabrication of flexible sensor devices and soft circuits on a variety of flexible substrates (Figure 2c,d) [35,39,44]. Importantly, the particles wrapped by the oxide can be easily wetted by the liquid metal itself (Figure 2e), and swallowing oxide-encapsulated particles can adjust the properties of liquid metal. For example, the mixture consisting of liquid metal and tungsten (W) particles exhibits two- to threefold enhanced thermal conductivity [45]. Overall, the oxide of liquid metal can be used as a detection agent for gas sensors, and could also help liquid metal realize the preparation of sensor structures or circuits.

### 2.3. Electrical Properties of Liquid Metal and Its Composite

**Figure 3:**
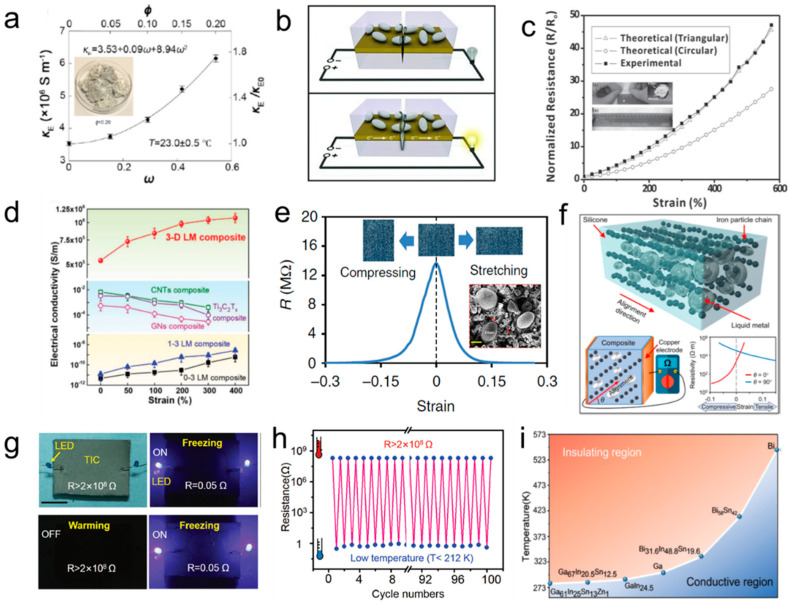
Electrical properties of liquid metal and its composite. (**a**) The enhanced electrical conductivity of liquid metal (LM) by mixing Cu particles [47]. Copyright (2017) American Chemical Society. (**b**) The process of autonomic conductivity restoration of a circuit containing liquid metal particles [49]. Copyright (2012) WILEY-VCH. (**c**) The relationship between resistance and strain [53]. Copyright (2013) WILEY-VCH. (**d**) Electrical conductivity as a function of the strain of the composites [55]. Copyright (2020) WILEY-VCH. (**e**) The resistance-strain curve of the liquid metal-filled magnetorheological elastomer. Stretching and compression all enhance conductivity [56]. Copyright 2019, Springer Nature. (**f**) The liquid metal composites enhanced electrical anisotropy under deformation [57]. Copyright (2020) American Chemical Society. (**g**) The liquid metal-based composite becomes conductive after freezing and recovers to electrical insulative again after warming. (**h**) Resistance change between electrical insulative and conductive for 100 cycles under the temperature regulation [59]. Copyright (2019) WILEY-VCH. (**i**) The conductive and insulating regions based on the melting points of the liquid metal droplets dispersed in dimethicone [61]. Copyright (2019) American Chemical Society.

Liquid metal alloys with intrinsically soft characteristics and liquid metal composites have many unique electrical properties. The conductivity of a Ga-based liquid metal can reach 3.4 × 10^6^ S/m which is higher than most non-metallic materials [46]. Swallowing highly conductive particles by an electrochemically wetted method can improve its conductivity, however, the liquid metal mixture is still roughly 20 times less conductive than non-flexible copper (at 20 °C) (Figure 3a) [47]. Hence, liquid metal should be considered for applications (including the biosensors) in which its soft nature, high conductivity, and some unique qualities (such as the strain-enhanced conductivity) are required [48].

Biosensors containing a liquid metal circuit can exhibit high electronic robustness because of its low melting point and fluid characteristics. For example, liquid metal particles covered with urea-formaldehyde with a mean diameter of 10 μm were utilized to restore conductivity in a broken circuit by the release its liquid core (liquid metal) to the damage site (Figure 3b) [49]. The circuit containing liquid metal microparticles can also heal spontaneously. When damaged, the droplets rupture to form new connections with neighbours and reroute electrical signals without interruption [50,51,52].

Soft liquid metal can undergo large deformations while maintaining its electrical stability and continuity. Thus, compositing with highly flexible organic materials can fabricate a highly stretchable conductor. Generally, the resistance of bulk liquid metal changes linearly with the value of deformation and satisfies the formula R=ρL/S  (Figure 3c) [53]. However, the composites consisting of liquid metal micro/nano particles may demonstrate the stretch-enhanced conductivity [54]. For example, Wang et al. reported that the electrical conductivity of the 3D LM composite increases monotonically with applied tensile strain, from 5.3 × 10^5^ S/m under the stretch-free condition to 1.1 × 10^6^ S/m at 400% strain (Figure 3d) [55]. Similarly, a liquid metal-filled magnetorheological elastomer shows the minimum conductivity in the relaxed state and increases drastically under any deformation (Figure 3e) [56]. Moreover, based on the advantages of flexibility and high conductivity, liquid metal-based composites with electrical anisotropy have also been discovered. Li et al. proposed a composite that exhibits anisotropic conductivity and a significantly enhanced electrical anisotropy under mechanical deformation (Figure 3f) [57]. Those provide new ideas for the preparation of high-strain biosensors [58].

Besides, liquid metal composites also demonstrate a reversible transition between the insulator and the conductor (resistivity span more than 10^9^ times) through temperature regulation alone (Figure 3g,h) [59,60]. Furthermore, Liu et al. made temperature tunable conductor–insulator transition liquid metal composites in a diverse range. According to the melting point of liquid metal, they further roughly determined the conductive and insulating regions to guide future specific applications (Figure 3i) [61]. Those composites show the potential for temperature sensing in extreme environments.

It has not been found that room temperature liquid metals have a piezoelectric effect. So far, liquid metal-based mechanical sensors are realized by measuring the change value of resistance [62] and/or capacitance [63,64]. For example, liquid metal strain sensors can sense strain by measuring the resistance change resulting from the deformation of the liquid metal circuit during elongation [65]. A clear correspondence between deformation amount and the change of resistance/capacitance, such as an obvious linear relationship, is the basic condition for high-precision sensing. Thus, the reliable electrical properties of liquid metals and their composites described above are highly dependent on sensors. We will examine these in detail in Section 3.

### 2.4. Biocompatibility

Biocompatibility, an important factor for biosensors, must also be considered. Unlike mercury, Ga and its alloy remain stable at room temperature and do not volatilize because of the lower surface vapor pressure. This allows gallium and its alloy to be used stably in a normal temperature and pressure environment. Although the toxicity of liquid metal remains controversial, there is increasing evidence showing its biocompatibility both in vitro and in vivo. Small doses of bulk liquid metal have been proven to be biocompatible. For example, gallium alloys have been employed as implantable biomaterials, such as restorative material in dentistry and as reconnection agents for nerve injury and bone cement [66,67]. In addition, liquid metals entering the body’s circulatory system also display good biocompatibility. Lu et al. systematically evaluated the toxicity of liquid metal particles in mice, and none of the important indices of liver function, including alanine aminotransferase, aspartate aminotransferase, alkaline phosphatase, and albumin concentration, were affected by the intravenous injection of liquid metal particles [68]. The mechanism of low toxicity for biological bodies remains elusive but appears to be strongly related to the following factors. One is that the liquid metal particles can be gradually degraded when exposed to an acidic environment. The other factor is that residual liquid metal (perhaps the majority) can be removed through both fecal and renal excretions [25]. For most liquid metal-based biosensors, the embedded structure prevents the liquid metal from directly contactingthe tissues. Overall, liquid metal can be used as a safe material to construct biosensors.

## 3. Applications

**Figure 4:**
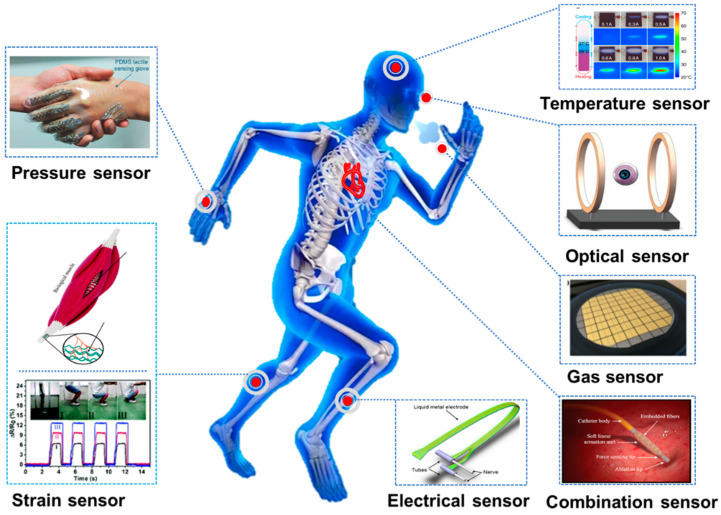
Applications of biosensors based on liquid metal.

Liquid metal is a favorable candidate as a transducing element for in vivo or in vitro signals in biosensors due to its outstanding features. Attributed to its high electrical conductivity, liquid metal has been widely used as soft electrodes and electrical interconnection. As is shown in Figure 4, owning to their high flexibility and adhesivity, the on-skin biosensors based on liquid metal are able to sense in vitro signals dynamically and conformally, such as temperature, pressure, strain, gas, and light. Besides, it is possible to utilize liquid metal in implantable sensors due to its inherent biocompatibility, for example, artificial muscle, nerve connection, and implantable sensors.

### 3.1. Mechanical Sensor

**Figure 5:**
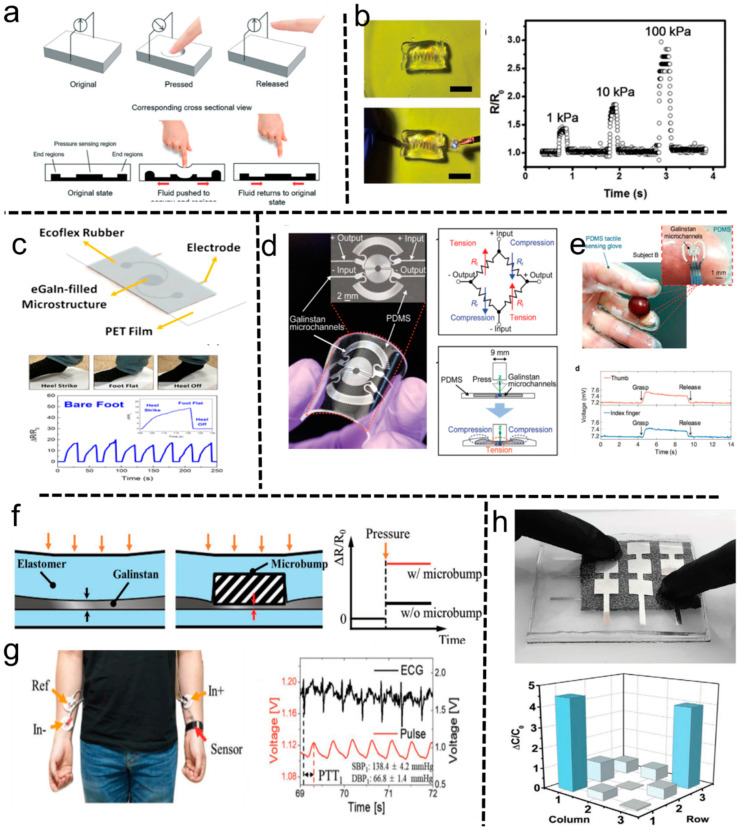
Liquid metal–based pressure and tactile sensors. (**a**) Schematic diagram of a liquid metal resistive pressure sensor [71]. Permission from The Royal Society of Chemistry (RSC) under a Creative Commons Attribution-Noncommercial 3.0 Unported Licence. (**b**) The optical picture and sensing performance of the 3D helical LM circuit/hydrogel matrix sensor [72]. Copyright (2018) WILEY-VCH. (**c**) The pressure sensor consisting of Ecoflex and EGaIn. The characteristic electrical responses of the tactile sensor when subjected to dynamic loading and unloading cycles of barefoot stepping [73]. Copyright (2016) American Chemical Society. (**d**) Microfluidic tactile diaphragm pressure sensor with liquid metal Wheatstone bridge circuit. (**e**) The real-time response was recorded from sensing the process of grasping a grape [74]. Copyright (2017) WILEY-VCH. (**f**) Effect of the microbump on pressure sensitivity. (**g**) Blood pressure monitoring system using the pressure sensor consisting of microbump [23]. Copyright (2019) WILEY-VCH. (**h**) The tactile sensor array is based on liquid metal elastomer foam and its capacitance change ratio [75]. Copyright (2020) WILEY-VCH.

Biomechanical movement is achieved through force generated by muscle stretching or contraction. Perceiving the environment by touch (i.e., pressure) is also achieved through mechanical interaction. Therefore, the mechanical sensors for pressure, tension, and strain are an important part of detecting biological motion and health. Liquid metal mechanical sensors are mainly realized by measuring the change in resistance or capacitance. The principle of resistive/capacitance mechanical sensors is based on the measurement of the change to an electrical signal induced by the deformation of liquid metal under mechanical force. The change value of resistive can be calculated by R=ρL/S, where ρ is the resistivity, R is the resistance, S is the cross-sectional area, and L is the length. The intrinsically low Young’s modulus is obtained duet to the liquid state of Ga and its alloy at room temperature. Thus, mechanical excitation/stress/strain/pressure can easily cause the deformation of the liquid metal circuit, which further induces the electronic signal change. Once the external force acting on the surface of the sensor stimulates the deformation of liquid metal, the corresponding mechanical signal can be calculated by the detectable electrical signal (principle depicted in Figure 5a). The sensitivity of the sensor depends not only on the liquid metal, but also on the encapsulating organic materials. A liquid metal with a low modulus can be the core component of a highly responsive sensor. However, the sensitivity of the sensor depends not only on the liquid metal, but also on the encapsulating organic materials. Thus, to improve the overall performance of sensors, the packaging material with low Young’s modulus is highly desirable [69,70]. The value of capacity can be calculated by $C=\varepsilon_{0}\varepsilon_{r}Ad$, where *C* is the capacitance, *ε_0_* is the vacuum permittivity, *ε_r_* is the relative permittivity, *A* is the area of electrodes, and *d* is the distance between electrodes. Generally, these sensors consist of two electrodes separated by a dielectric. The distance between two electrodes of the capacitor decreases under pressing or stretching, and the parameter, generally the distance *d* and the relative permittivity *ε_r_*, decreases correspondingly. By measuring the change of capacitance, the mechanical signal can be obtained.

#### 3.1.1. Pressure Sensor

Liquid metals with low modulus easily deform when force is applied and are highly sensitive to mechanical signals. As mentioned above, liquid metal does not show a piezoelectric effect, making liquid metal sensors limited to resistive and capacitive. Micro-deformation on the pressure sensor leads to fluid displacement or deformation of liquid metal which corresponds to the alteration in resistance or capacitive. By characterizing those electrical properties changes, highly sensitive and flexible sensors can be prepared (Figure 5a) [71]. Those pressure sensors are capable of resolving small pressure changes in the few kPa range, making them suitable for applications such as tactile sensing and heart-rate monitoring. Compared with that of solid materials, the pressure to deform flexible liquid metal is smaller. Meanwhile, the structure of the liquid metal circuit is easier to design and fabricate by using micro-channel infusion, or liquid metal printing.

Generally, the resistance pressure sensors perform through sensing the monitored resistance change of liquid metal circuits induced by the simultaneous deformation of liquid metal and matrix materials. Thus, the performance sensors mainly depend on the sensitivity of the matrix material and the liquid metal to pressure. Soft materials with low elastic modulus and the functional liquid metal circuit could construct highly sensitive pressure sensor, and even realize tactile sensing. A 3D helical LM layout incorporated inside the hydrogel matrix was used to fabricate soft pressure sensors. Limited by the high modulus of the hydrogel, the sensor presents a poor performance in distinguishing varying levels of pressure (i.e., 1, 10, and 100 kPa) (Figure 5b) [72]. The 3D liquid metal circuit can theoretically increase sensitivity, but it seems that the impact of the 3D structure is not as evident as that of low elastic modulus. The matrix material with lower modulus, such as soft elastomeric silicone rubber and PDMS, can achieve high-precision pressure sensing. Lim et al. proposed a sensor with a 2D liquid metal circuit that could reach high sensitivity (2–20 × 10^−3^ kPa^−1^) and be capable of distinguishing compressive loads with an extremely large range of pressure (2 to 400 kPa). It is able to distinguish and quantify the localized pressure exerted through distinct actions like barefoot stepping and walking using different footwear (Figure 5c) [71,73]. Optimizing the liquid metal circuit can improve the accuracy of the sensor and reduce the response time. For example, an embedded equivalent Wheatstone bridge circuit makes the most of tangential and radial strain fields, leading to high sensitivities of a 0.0835 kPa^−1^ change in output voltage. Galinstan microchannels capable of resolving sub-50 Pa changes in pressure with sub-100 Pa detection limits and a response time of 90 ms is demonstrated to detect subtle pressures with a detection limit of ≈98 Pa and resolution of less than 50 Pa (Figure 5d,e) [74]. Generally, the force will be buffered by the organic matrix material, even with low modulus, when the liquid metal circuit is far away from the stress point. Using microstructure to transfer stress is another way to improve the performance of sensors (comparing Figure 5a,f). An LM-based pressure sensor with a 3D-printed rigid micro-bump array obtains enhanced sensitivity (0.158 kPa^−1^) by locally concentrating the deformation of the microchannel without obvious hysteresis and a stable signal response under cyclic loading (Figure 5f,g) [23].

Similarly, the capacitive changes once the liquid metal circuit acted by vertical pressure will deform [76]. Compared with resistive sensors, capacitive sensors show better sensitivity and can be simpler and more stable. The sensitivities of the GaIn-BiInSn leakage-free electrodes based sensors can reach 0.45 MPa^−1^ [77]. Sensitivity to a given force is maximized by using dielectric materials that are soft and have a high dielectric constant. Ecoflex is a better candidate. Yang et al. introduced a liquid metal-based composite consisting of EGaIn and Ecoflex for pressure sensing, called liquid metal elastomer foam [75]. By creating a foam architecture, composite obtains large permittivity, large permittivity change, and is ultra-soft. Pressing the sensor gave rise to an increase in permittivity. After the air had been fully pressed out, the change in permittivity would become negative due to the changed shape of EGaIn droplets. These properties gave the composite the ability to be applied in tactile sensing and motion sensing (Figure 5h) [75]. Interestingly, they also achieved to power tactile sensor wirelessly from 3 m away with high power conversion efficiency.

Overall, liquid metal pressure sensors have various applications such as e-skins [78] and soft keyboards [79], and ECG monitoring [80], etc. Liquid metal made sensors and other electronics can be integrated onto clothes and accessories, such as shirts, socks, or wrist bands, to establish intelligent human–machine interaction, and health monitoring [39]. For real-life application convenience, integrated electronics were designed to be wireless when it is possible.

#### 3.1.2. Strain Sensor

Muscle contraction and muscle relaxation help in various physiological activities, such as joint motions and heartbeat. Dielectric Elastomer Actuators (DEA), an artificial muscle structure, is a typical sandwich structure combing a dielectric elastomer and soft electrodes on both sides of it. Compliant electrodes should not only be super stretchable with negligible stiffness but also highly conductive at high strains [81]. Liquid metal can be adopted as soft electrodes to actuate certain elastomer to fabricate artificial muscle, having superior performance to other traditional electrodes.

Relatively good compliance and conductivity are provided by carbon-based (carbon grease, graphene [82], and single-walled carbon nanotubes [83]), and metal-based electrodes [84], while the intrinsic stiffness of solid confines the expansion of the elastomer middle layer. For conductive liquid electrodes [85], high fluidity, poor conductivity, and easy electrolyzation impede its further practice. Semi-solid electrodes, including carbon grease and silver grease, are a better compromise choice compared with solid or liquid electrodes on stiffness and conductivity, but conversely, have unstable conductivity due to elements aggregation while deformation. Hence, Liu [86] proposed a liquid metal electrode with high conductivity and negligible stiffness at high strain simultaneously, promising large area strain at relatively lower voltage and stability under 30 cycling tests. Clearly, liquid metal electrode-a single-layer compliant electrode of DEA is fabricated simply by painting liquid metal on the 300% biaxially prestrained VHB 4905 film directly. Area expansions induced applied voltage of the films were measured to evaluate the performance of such liquid metal electrode. The result shows that the DEA achieved a much better strain of over 250% at 3.5 kV than the existing approaches. However, the sample underwent an electric breakdown due to the defects of the DE films, and the multi-layer structure remains to be explored in the future base on liquid metal electrodes.

Some actuators have been introduced in pneumatic artificial muscles (PAMs) based on their compliant, light, and compact features. A pressure sensing EGaIn microchannel is currently used to investigate the force sensing in the actuator [87]. A novel concept of a 3D helical soft sensor positioned around McKibben’s muscle was introduced by King and Park et al. [88,89]. To improve the non-linear response of previous actuators, Park et al. [89] designed a McKibben PAM comprising a multi-layered elastomer tube with embedded Kevlar threads and an EGaIn filled spiral microchannel (diameter of 120 μm). Under air pressure of 0–105 kPa., the prototype was able to create linear contraction force and strain up to approximately 65 N and 22.5%, respectively.

**Figure 6:**
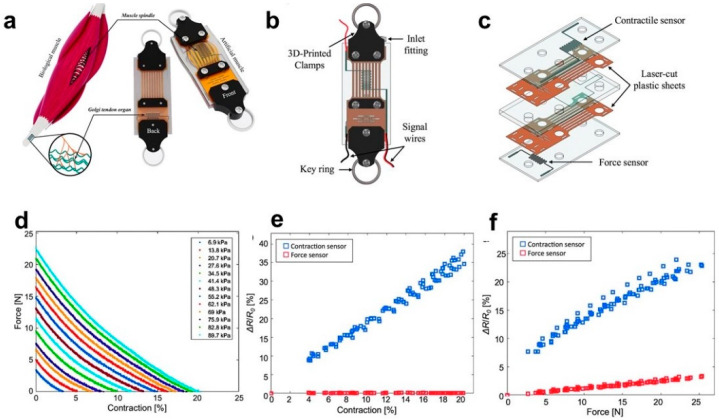
Typical models of artificial muscle and its sensor characteristics. (**a**) Schematic comparison of sFPAM (sensorized, flat, pneumatic artificial muscle) with biological muscle. (**b**) Illustration of the completed actuator at rest with labels designating key components. (**c**) Exploded view provides a clear visualization of the contraction and pressure sensors. (**d**) Force–contraction relationship at pressures ranging from 0 to 90 kPa. (**e**) The resistive response of force and contractile sensors due to contraction and (**f**) force [90]. Copyright (2019) Jackson Wirekoh et al., Published by Mary Ann Liebert, Inc.

Besides the McKibben’s muscles, a multi-layer pneumatic artificial muscle embedded with biomimetic microfluidic sensors based on the muscle spindles and Golgi tendon organs (Figure 6a) was proposed as well [90]. The structure of the actuator was stacked with layers and core in the following order: silicone layer (force sensor), laser-cut plastic, sheet, core, laser-cut plastic sheet, and silicone layer (contractile sensor) (Figure 6b,c). The top and bottom layers of silicone were embedded with microchannels (sensors) that have cross-sectional dimensions of 0.2 × 0.2 mm thickness, in which liquid metal EGaIn was injected to act as the resistive force and contractive sensors, mimicking the function of the Golgi tendon organ and muscle spindle in humans.

The force versus contraction response characterization was tested by a repeating procedure. In the substantial process, the force of the program kept increasing with the constant pressure applied, in which the pressure was determined by air volume. The sFPAM was then manually contracted at a speed of 10 mm/min at a steady-state value of force until the program’s force reading returned to zero. The corresponding contraction and force change responses to the pressure (Figure 6d–f) were in a range of 0–90 kPa, with a maximum value of 19.8% ± 0.2% and 24 ± 1.37 N respectively. It is noteworthy that the desirable decoupling effect of contractile and force sensors will facilitate effective control in the practice. The integrated application of such actuators in soft robots needs to be further developed.

Artificial muscles can be regarded as strain sensors in vivo. Moreover, the strain-sensing used to monitor the deformation and the tensile force in vitro are significant for the detection of biological vital signs. Strain sensors can be made by embedding liquid metal in an organic matrix [53]. Strain induces the shape change of liquid metal that maintains electrical stability and continuity under deformation. Thus, the amount of change in resistance and/or capacitance corresponds linearly to that of deformation. By measuring the resistance and/or capacitance change induced by shape change, the value of strain can be obtained [91].

**Figure 7:**
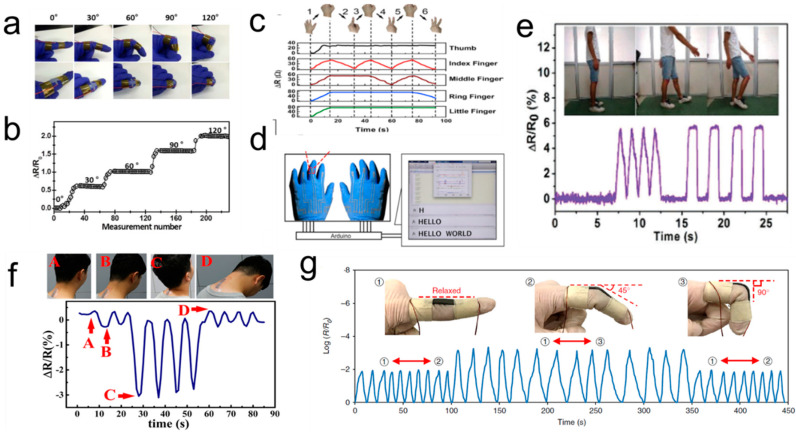
Liquid metal-based strain sensors. (**a**,**b**) Finger motion detection using strain sensors and its corresponding resistance change [95]. Copyright (2019) American Chemical Society. (**c**) Motion monitoring of five fingers with slow bending and straightening. (**d**) Use wearable gloves for typing “HELLO WORLD” [96]. Copyright (2018) the author(s). (**e**) A highly flexible strain sensor monitors knee motion [62]. Copyright (2013) Royal Society of Chemistry. (**f**) Real-time monitoring of necks motions by strain sensor consisting of liquid metal micro particles [95]. Copyright (2019) American Chemical Society. (**g**) Unusual resistance changes of the strain sensors under cyclic bending [56]. Copyright (2019) Springer Nature.

Liquid metal-embedded elastomer compositions have been widely investigated for strain sensors [63,92]. Human motion can be monitored by attaching strain sensors on human joints, such as fingers [93], wrists [94], and necks [95]. Figure 7a, b presents the resistance change when the finger is bent at different angles, with different signal peaks representing different bending degrees. Therefore, special electrical signals can be generated through the whole palm detection, further realizing simple logic operations, such as typing “HELLO WORLD” (Figure 7c,d) [96]. Similarly, liquid metal is filled into the core of the hollow and extremely stretchable elastomeric fiber, and the resulting fibers are used to fabricate the high strain sensors to detect the movement of large joints, such as the knee (Figure 7e) [62,64]. The maximum stretch of those sensors is not limited by the breaking elongation of liquid metal. Theoretically, those sensors can obtain an ultra-wide measurement range of strain (up to ~700%) [53].

In addition, liquid metal micro/nano particles are also used to replace traditional solid particles to prepare flexible sensors [97]. For example, EGaIn in combination with sonication was able to initiate the polymerization of acrylic acid and further gel the resultant poly(acrylic acid) into self-healing strain sensors [98]. The hydrogel sensor exhibits excellent electrical and mechanical self-healing ability and can be fabricated as an epidermal sensor, which can accurately monitor human activities, such as necks (Figure 7f) [50,95]. As mentioned above, liquid metal composites demonstrate strain enhanced electronic conductivity. In combination with a highly flexible matrix, liquid metal-based composites have the potential to be applied for developing highly sensitive flexible sensors. As is shown in Figure 7g, the resistance decreased drastically by more than 3 orders of magnitude when the bending angle increased to 90° [56].

In Section 3.1, we reviewed the liquid metal-based pressure and strain biosensors, and compared their performance and evaluation in Table 1. Due to the unique physical and electrical properties of liquid metal, they have shown many advantages in flexible and stretchable biosensors. Artificial muscle is one of the important applications. However, the accuracy and response time of liquid metal sensors need to be improved. Therefore, more compatible composite materials and optimal microstructure still need to be explored.

### 3.2. Gas Sensor

**Figure 8:**
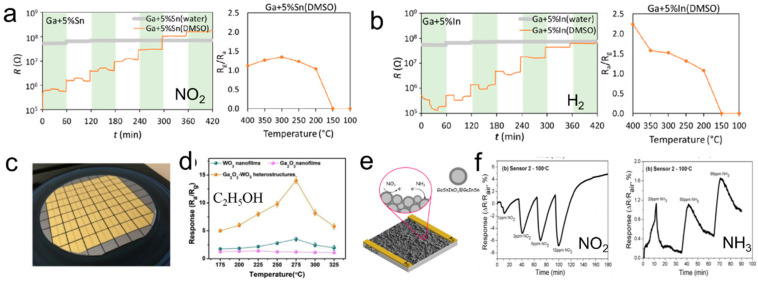
Liquid metal-based gas sensors. (**a**) The response of the sensors to NO_2_ gas. (**b**) The response of the sensors to H_2_ gas [22]. Copyright (2020) American Chemical Society. (**c**) The optical image of WO_3_-Ga_2_O_3_ sensors. (**d**) The resistance change of WO_3_-Ga_2_O_3_ sensors at 100 ppm ethanol under the different working temperatures [101]. Copyright (2019) Elsevier B.V. All rights reserved. (**e**) Schematic diagram of low-temperature gas sensing. (**f**) The response of low-temperature gas sensing to NO_2_ and NH_3_ [102]. Copyright (2017) Elsevier Ltd.

The gas sensor is an indispensable part of constructing a biological robot because the recognition and perception of gas are one of the necessary abilities for living organisms. The metal oxide semiconductor sensor can detect the gas by changing its conductivity after adsorbing the gas. As is mentioned above, β-Ga_2_O_3_ is a semiconductor material with a wide bandgap and high breakdown electric field [41]. The conductivity of β-Ga_2_O_3_ can be easily influenced by an ambient atmosphere at elevated temperature. However, at low temperatures, the oxygen-vacancies diffusion is frozen and the bulk electrical conductivity no longer responds to the change of circumstance gas composition [42]. Based on this principle, the liquid metal gas sensors responding to CO and H_2_ have been developed [99,100]. Recently, Kourosh et al. proposed a novel gas sensor by operating liquid metal micro/nano particles after annealing to detect the nitrogen dioxide (NO_2_) and hydrogen (H_2_) (Figure 8a,b). The NO_2_-sensing mechanism is based on the chemisorption of NO_2_ gas molecules onto the sample surface. Differently, the H_2_-sensing mechanism is described as the n-type behavior of liquid metal oxide. The chemical interactions between H_2_ gas molecules and oxygen ions on the sample surface at elevated temperatures. The number of free electrons is reduced, which results in an increase of the resistance in sensor materials. The responsivity of liquid metal oxide compounds is achieved through the choice of solvent (DMSO or water) and added elements (In, Sn, or Zn). The Ga–In sensor only presents a response to NO_2_ (4.5 ppm) at 150 °C but shows a higher selectivity toward H_2_ (1.0%) when the temperature ramps up to 350 °C and above. A similar trend is observed for a Ga only compounds selectivity to H_2_ above 300 °C. The preparation process in DMSO gave rise to predominantly monoclinic β-Ga_2_O_3_ crystals which are favorable for gas sensing, while the emergence of rhombohedral α-Ga_2_O_3_ phases from the water sonication process led to inactive samples [22]. Overall, β-Ga_2_O_3_ is also an efficient usage as sensing materials for inorganic gas. Recently, organic gas sensors based on β-Ga_2_O_3_ was developed. Serge et al. proposed a C_2_H_5_OH sensor consisting of Ga_2_O_3_-WO_3_ heterostructures with a thickness of approximately ~8.0 nm. This sensor can make repeatable and reversible responses to C_2_H_5_OH in the concentration range of 1–600 ppm. The resistances of Ga_2_O_3_-WO_3_ heterostructures decrease from 6.5 MΩ to 0.4 MΩ in 3s after exposure to ethanol, exhibiting the n-type behavior. This gas sensor exhibited about 4 and 10-fold improvement to that of WO_3_ and Ga_2_O_3_ nanofilms at 275 °C. Besides, shorter response/recover time and excellent selectivity towards ethanol were observed (Figure 8c,d) [101].

It is obvious that the working temperature of these gas sensors is high (almost above 200 °C). Reducing the temperature is a significant research aim regarding oxide semiconductor sensors. Recently, physisorption between Ga oxide and inorganic gas at 100 °C was discovered. Based on this phenomenon, a low-temperature gas sensing with a detection limit as low as 1 and 20 ppm for NO_2_ and NH_3_ respectively was fabricated (Figure 8e,f) [102]. The resistance of the sensor decreased when exposed to NO_2_ gas due to the formation of negatively charged oxygen species. Moreover, the opposite resistance change is observed upon exposure to NH_3_. Although this sensor shows poor durability and does not fully recover to the original baseline, this new mechanism provides another way for low-temperature gas sensing.

Gas sensors based on liquid metal semiconductors are an emerging way to detect gases, including CO, H_2_, NO_2_, C_2_H_5_OH, and NH_3_. We summarized the current indicators of liquid metal gas sensors in Table 2. Obviously, the detection range of the gas sensor based on liquid metal-semiconductor is small, and it is difficult to simultaneously detect multiple types of gases. In addition, for certain inorganic gases, such as H_2_, the detectable threshold concentration is higher. Although it is currently possible to achieve gas detection at 100 °C, there is still a long way to go for high-precision room temperature gas detection.

### 3.3. Temperature Sensor

Temperature is an important parameter for describing the life characteristics of organisms. The volume expansion method and the Seebeck effect are the principles of common temperature sensors. The volume expansion of fluid could be determined by the dimensions of the enclosing container and is indirectly correlated to a specific temperature value. Thus, the measured temperature can be determined by detecting the volume change of liquid metal. The traditional watermark thermometer is based on a similar mechanism. There are commercially available gallium-based volume expansion thermometers because of their low toxicity. Different from mercury, Ga is not easy to volatilize and can hardly be inhaled by the human body in the atmospheric environment. Besides, it is proven to have low toxicity even if it enters into the body’s circulatory system. However, the melting point of gallium (29 °C) is higher than that of mercury (−39 °C), and it is difficult to obtain the Ga-based alloy with an ultra-low melting point. Moreover, gallium alloys have a negative coefficient of thermal expansion. Once the temperature is lower than its melting point, the gallium-based thermometer may cause structural damage due to the negative expansion. Therefore, Ga-based thermometers are not currently suitable for low-temperature measurements. Besides, the sensitivity of volume expansion-based temperature sensors is low.

**Figure 9:**
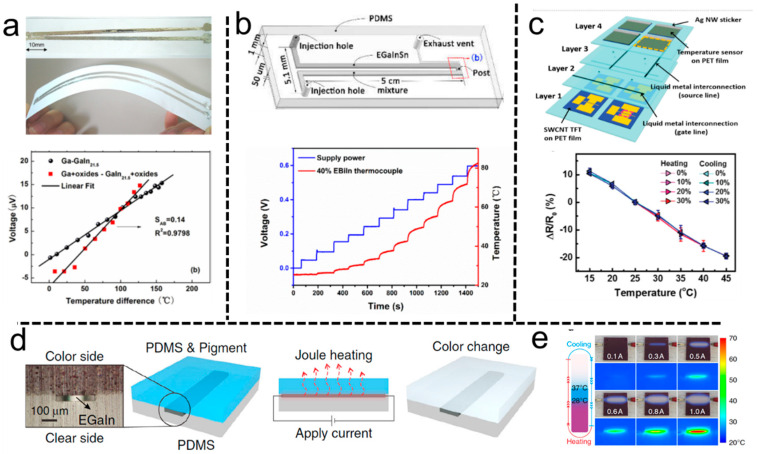
Liquid metal temperature sensors. (**a**) The top is the Ga based thermocouple written on the paper. The bottom is the measured thermoelectric voltage as a function of temperature difference [3]. Rights managed by AIP Publishing. (**b**) Schematic of micro-thermocouple; One channel was filled with EGaInSn, and the other was filled with a low-melting-point Bi-based metal alloy mixture. The bottom shows the relationship between supply power and temperature [103]. Copyright (2019) by the authors. (**c**) Schematic of the stretchable temperature sensor array consisting of the liquid metal circuit. The resistance change of the sensor at a different temperature under a biaxial strain of up to 30% [106]. Copyright (2016) WILEY-VCH. (**d**) Schematic of the color displayed sensor consisting of a liquid metal circuit between two layers of PDMS. Joule heating changes the color of the pigment in the PDMS. (**e**) The sensor with blue and red pigments that alter color in response to heat [65]. Copyright (2019) Springer Nature.

Thermocouples based on the Seebeck effect are commonly used temperature measurement devices in the industry. Those sensors are more sensitive and respond more quickly. Liu et al. proposed the direct printing of thermocouples by liquid metal [3]. The fabricated temperature sensor composed of gallium and its matching metal exhibited excellent linear dependence between thermoelectric voltage and temperature within the range of 0 to 200 °C. Figure 9a presents the voltage-temperature difference profiles of the thermocouples consisting of Ga and the matching metal wires. All these thermocouples exhibit nearly linear behavior in the experimented temperature range. The accuracy of the thermocouples was ±0.5 °C. Further, it was disclosed that liquid metals with high purity could be used for high precision thermocouples with tiny size, which were quite convenient for microchannel measurement due to their fluidity in making sensors. Further, a low-melting-point Ga-Bi alloy micro-thermocouple was proposed by using a microfluidic injection method. This thermocouple shows a higher Seebeck coefficient (−10.54 μV/K) and high flexibility. No structural damage and electrical degradation were observed after 200 times bending at 90° (Figure 9b) [103].

Although many soft temperature sensors have been reported, stretchable temperature sensors maintaining a stable performance under 50% strain are still challenging [104]. An obvious limitation is that the embedded circuit is easily broken under deformation [105]. As mentioned above, severe deformation, such as bending and stretching, will not affect the electrical stability and the connection of the liquid metal circuit. Based on this principle, a stretchable temperature sensor with an active-matrix consisting of EGaInSn and single-walled carbon nanotubes was fabricated. These highly stretchable temperature sensors exhibit a high resistance sensitivity of 1.0% °C^−1^ and a response time of 1.8 s in the temperature range from 15 to 45 °C (Figure 9c) [106].

The above-mentioned temperature sensors usually measure electrical changes to identify temperature changes. We compared the above-mentioned sensors in Table 3. The temperature information needs to be processed by the central arithmetic unit to realize the corresponding logic control. This is similar to the complex reflexes of organisms to external temperature stimuli. Of note, simple reflection systems similar to living organisms can also be realized by liquid metal logic circuits. Dickey et al. proposed a soft liquid metal-based sensor to achieve a simple reflection of tactile logic [65]. Liquid metal is patterned between two layers of PDMS, one transparent and one containing thermochromic species (Figure 9d). Passing a current through the liquid metal generates Joule heating and the thermochromic species alter their colors above critical temperatures due to the molecular structure rearrangement (Figure 9e). Deformation can affect the resistance of the liquid metal circuit, further affecting the Joule heating. Thus, the deformation of liquid metal will couple shape changes to Joule heating, thereby enabling the tunable thermo-mechanochromic sensing of touch and strain.

### 3.4. Optical Sensor

Either solid and liquid nanoparticles [107,108] or colloidal liquid gallium-based nanoparticles [109] have been verified to have typical UV plasmonic properties. Recently, attention has been focused on the applications that integrate optical UV plasmonic with non-optical properties like electronics, photoacoustic [110], and optomechanics [111], and electrochemistry [112] at the nanoscale.

**Figure 10:**
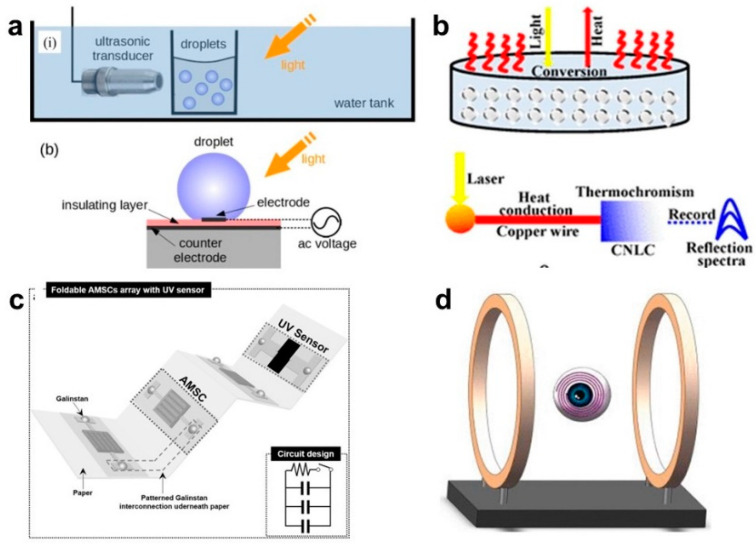
Liquid metal–based optical sensors. (**a**) Schematic illustration of experimental setups for the excitation of capillary oscillation of nanodroplets with ultrasound (top) and AC voltage (bottom) [110]. Copyright (2017) American Physical Society. (**b**) LM microspheres are used as a free-standing photothermal medium [113]. Copyright (2019) American Chemical Society. (**c**) Schematic illustration of a foldable AMSCs array with a UV sensor on a single liquid metal patterned paper substrate [114]. Copyright (2017) WILEY-VCH. (**d**). The geometric model of a 2-D liquid metal induction coil: Helmholtz Coil system with the induction coil inside [116]. Copyright (2018) by the authors.

Ivan et al. [110] considered the dynamically reconfigurable plasmonic structure of liquid metal through experimental equipment employed in photoacoustics. The ultrasonic transducer and light showed in the schematic figure (Figure 10a) facilitate the capillary oscillations of the nanodroplets to manipulate the plasmon resonances, allowing it to operate in in vivo biological environments and opening new opportunities for biology and medicine.

Taking the equivalent optical property of LM microdroplets into account, Lin et al. [113] applied the microdroplets to make optical sensors with linear response and to conduct the solar energy harvesting based on the intrinsic photothermal property of them. In their experiment, monodispersed LM microspheres can simply be fabricated by a size controllable microfluidic method. The photothermal effect induced NIR sensor with thermochromic chiral nematic liquid crystals as an endothermic medium can reflect blue color once receiving light from NIR radiation (Figure 10b). The reflectance performs a linear increase versus radiation time.

Combing the properties of flexibility, low melting point, and relatively high electrical conductivity, liquid metal facilitates the further investigation of promising optical sensors. Yun [114] proposed a high-performance UV sensor system driven by an integrated energy storage system. With the patterned liquid metal interconnections, the device integrated with a UV sensor and asymmetric micro-supercapacitors is likely to be stretchable, flexible, and foldable (Figure 10c). The integrated device showed excellent mechanical stability under over 2000 folding cycles on a patterned, waterproof mineral paper with liquid metal Galinstan. Besides, the sensor was operated for 1500 s with repetitive UV exposure cycles and folding deformation. Moreover, the asymmetric micro-supercapacitors can also provide energy without external wire-connection, guaranteeing the further application in wireless wearable and foldable devices embedded power sources.

Innovatively, wireless power transfer is playing a more and more significant role in biomedical implantable devices, thus attracting increasing attention in applications [115,116]. Amit et al. [115] put up with a wireless telemetry system based on EGaIn coils for an artificial retina to restore partial vision to the blind. The evaluating power transfer efficiency and stable performance imply the possibility of replacing the stiff and uncomfortable traditional coils used in biomedical implants. Then, Zhao et al. [116] further realized eye movement tracking by replacing traditional copper coils with the liquid metal coil (Figure 10d). The measured reliable performance of the LM coil guaranteed the promising applicable value in the implanted system combing with the intrinsic flexibility.

### 3.5. Electrical Sensor

**Figure 11:**
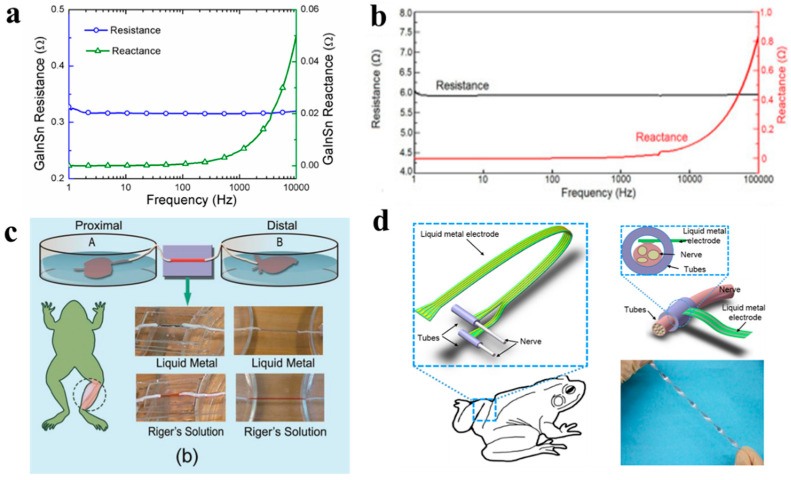
Applications in nerve connection. (**a**) The impedance curve of the GaInSn. Copyright (2014) by the authors [117]. (**b**) The impedance of gallium for signals with different frequencies [66]. Copyright (2016) Science China Press. Published by Elsevier B.V. (**c**) The schematic diagram of the transected sciatic nerve reconnected by liquid metal and Riger’s Solution, respectively [117]. Copyright (2014) by the authors. (**d**). The liquid metal nerve electrodes and the machining process [120]. Copyright (2017) by the authors.

Liquid metal is an al biomaterial for nerve connection that contributed to its high electrical conductivity, electrical stability, favorable compliance, and flexibility. Tests of electrical characteristic show that GaInSn alloy has a distinctive advantage over Riger’s solution, as the resistance of it (4.396 × 10^−8^ Ω·m) is much lower than that of Riger’s solution (0.8709 Ω ·m) and the resistance is more stable from 1 Hz to10 kHz [117] (Figure 11a). Gallium has the same stable resistance of 5.92 Ω at the frequency a range 0–10 kHz, and 0.51 Ω m at a temperature of −16~50 °C within the range of human activities [66] (Figure 11b). Both of the results verify the feasibility of LM for conducting electroneurographic signals and relative experiments on the bullfrog and mouse are conducted.

In the experiment of the electrical reconnection of the bullfrog’s sciatic nerve, liquid metal GaInSn was injected into a rectangular groove or glass capillary pipette to connect the extracted proximal sciatic nerve and gastrocnemius with the distal sciatic nerve [66] (Figure 11c). In the contrast experiments between LM and Ringer’s solution, the response of electrophysiological stimulation with LM as nerve connected medium was more similar to that of intact groups. Then, a further experiment employed gallium to treat the transected nerve in mice in vivo [30]. The output signal from the nerve reconnected shows a remarkably similar tendency to the intact nerve. Furthermore, the LM is found to improve the recovery of the nerve’s function. The muscle atrophy was observed to be successfully delayed by two months, which was obtained from the detection of fibrillation potential. The relieving effect of muscle atrophy was then validated in the histological analysis. Although the gastrocnemius exhibited pathological atrophied muscle fibers in the bot group, the LM treated nerve maintained a weight of 82% while the maintenance rate of the untreated group is 17%. The applications above exhibit significant value of the liquid metal in nerve repair. However, the trial above based on LM exhibits only nerve reconnection rather than nerve regeneration, which needs further investigations in the future [118].

The electrical stimulation of nerve connection relies on a rigid solid electrode to transient input and output signals, like silver-coated copper electrodes in previous research. However, the mismatch elastic modulus between soft tissues and the rigid electrode may impede the researcher from acquiring an accurate analysis and long-term application. Thus, researchers tend to develop a more soft electrode to facilitate compatible contact with tissues and potentiate the capability of nerve implants [119] (Figure 11d). The similar mechanical property between soft electrode and tissues (elastic modulus of 1.055 Mpa for LM, 0.1~1 Mpa for tissues) promises better integration and stable functionality. Guo [120] proposed a liquid metal-based flexible neural microelectrode array and applied it in recovering animal locomotion functions successfully. After evaluating the mechanical and electrochemical characteristics and biocompatibility of the soft electrodes, the microelectrode array was implanted into the nerve of the dead frog rhythmically contract and move of its lower limbs was achieved under electrical stimulation.

A series of experiments on neural connections proved the unique role of liquid metal. Further theoretical exploration is still necessary, but we should focus more attention on solving the problems faced by applications. For example, although liquid metal has been proven to have good biocompatibility, it is still unknown whether it will affect the surrounding tissues or organs when it exists in the body for a long time. At the same time, the problem of the sensitivity attenuation of liquid metal electrical signals may arise in the human body environment.

### 3.6. Multifunctional Sensor

**Figure 12:**
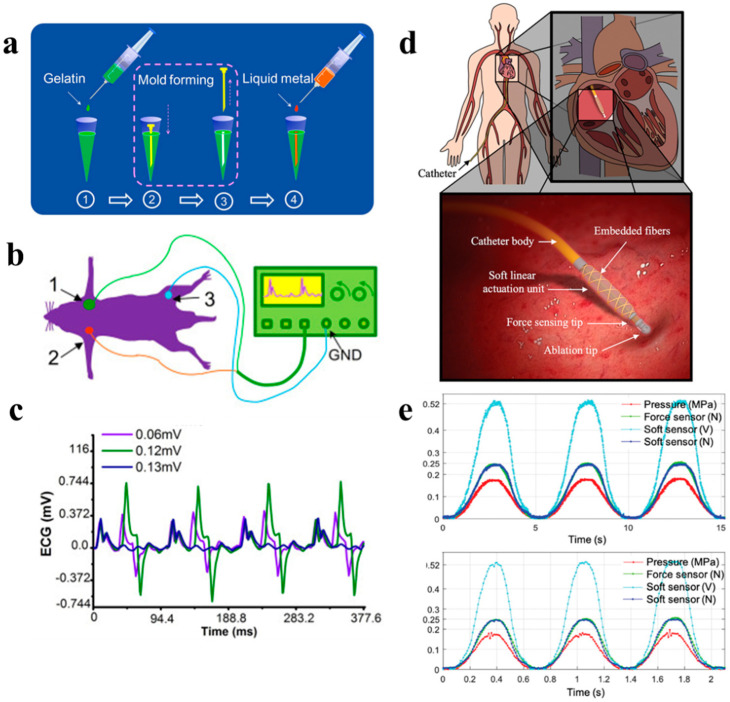
Implantable medical devices. (**a**) Alternative strategies for making implantable bio-electrode through directly injecting liquid metal and allied packaging material inside a pipette tip; (**b**) Schematics of in vivo experiment for measuring the mouse ECG signal using the injected electrode. (**c**) The recorded ECG signals of experimental mouse undergoing a 10 Hz electrical stimulation with different voltages [121]. Copyright (2019) Springer Nature. (**d**) Conceptual design of a cardiac ablation catheter integrated with soft actuation and sensing unit for dynamic force control. (**e**) The soft sensor response to sinusoidal input for dynamic characterization at 0.2 Hz and 1.5 Hz [122]. Copyright (2019) by the authors.

Implantable medical devices (IMDs) are efficient methods to monitor and treat serious diseases and physiological conditions. Concerning the typical problems—including invasive implantation surgery, related complications, high cost, discomfort, and long-term maintaining difficulty—of traditional IMD, Liu’s group first introduced liquid metal into the IMD field [121]. Based on a minimally invasive injection process, they fabricated 3D compliant medical electronics inside the biological tissues through directly injecting Ga_67_In_20.5_Sn_12.5_ into the target tissues (Figure 12a,b). With liquid metal embedded in packaging material (biodegradable gelatin), the new minimally invasion implantation brings less painfulness for patients compared with the conventional surgical principles. Besides, the advantage of simplicity and low cost makes liquid metal-based injectable electrodes outperform other IMD. The feasibility of the 3D electronics was demonstrated by in vitro and in vivo experiments. The results proved the excellent nerve connection property and good electrical properties of LM-based composite electrodes (Figure 12c). However, the precision of this manually-made electrode is expected to be higher through mechanical control in later research. Some more efficient packaging material can be an alternative to promise degradability and solidification.

Very recently, a compact and flexible implantable medical device was designed [122], integrating soft miniaturized actuation and sensing units with different sizes and material for cardiac catheters (Figure 12d). Both the soft elements are fabricated from eutectic gallium–indium (EGaIn). The in-situ actuation is achieved by a soft thick-walled cylindrical actuator and the innovative contribution of this work is that this soft fiber-reinforced actuator depends on hydraulic actuation rather than popular pneumatics [123], which further promise controllability and safety. The hyperplastic modeling of the designed soft sensing unit was given and provided theoretical parameterization and characterization of the unit. The following experimental validation presented the static and dynamic characterization of the miniaturized actuation and sensing unit. Corresponding to heart wall movement dynamics, the actuator is desired to keep stable at a displacement in the range of 1–2 mm. The unit can response with a ~0.52 V peak value at high frequencies of at least a bandwidth of 1.5 Hz, which satisfies the need in cardiac ablation procedures (Figure 12e).

## 4. Challenges

This review illustrated the achievements of liquid metal in biosensors. Liquid metal with typical characteristics––including electrical conductivity, flexibility, low toxicity, and low melting point––are currently used in diverse applications. This new-emerging biomaterial broadens the applications of biosensors in wearable and implantable devices. Despite the extensive progress that has been made in liquid metal biosensors, there are still several critical issues remaining to be tackled.

### 4.1. Materials and Fabrication

Materials used to be biosensors are relatively restricted in some specific types and alloys like GaIn, GaInSn, and BiInSn alloy. Considering the electrical conductivity, safety, and signal sensitivity, optimal compositions still needed to be explored. A strategy like modification seems to be practicable. For example, liquid metal particles are usually conjugated with receptor-mediated substances for targeted tumor ablation therapy [124]. Some specific surfactants are utilized to be anti-oxidation agents for oxide prevention in some circumstances. Thus, more modifying agents for liquid metal are considered to increase the bio-signal sensitivity by chemical reaction or bond formation. Feasible fabricating methods are necessary for the reliability of sensors. A high-resolution printing method based on Spraying and Wiping Processing is proposed by Liu’s group [125], the practice in SRR arrays with different feature sizes (2 mm and 1 mm) manifests the reliability for high-resolution circuits. The resolution of other flexible sensors and electrodes with smaller feature sizes based on liquid metal is expected to improve through this method. However, more high-precision and large-scale circuit preparation methods need to be explored.

### 4.2. Applications

For implantable biosensors, such as muscle, nerve connection, implantable medical devices, and optical sensors, it is necessary to take both the function and the patient’s experience into consideration. Liquid metal can be an ideal candidate for signal transmission in nerve reconnection. However, muscle atrophy in the tissue with the repaired nerve is inevitable, though the liquid metal alleviates and delays the necrosis time. Thus, great efforts should be made for promoting the regeneration of the transected nerve. It is expected to accelerate the research and clinical applications of nerve reconnection with the aid of a liquid metal electronic medium. On the other hand, concerning the feeling of the targeted treatment group, the implantable micro-medical device that needs to penetrate directly into the biological body is not always conformal in tissues. Generally, a hollow catheter is used to guide navigation. Hence, a soft and comfortable catheter is of vital importance for a comfortable experience and for facilities to lessen complications.

## 5. Perspective

Insight should be focused on sensors integrating monitoring, diagnosing, and therapeutic functions. Generally, most medical devices exert their influence independently. The in-situ or on-skin biosensors detect static signals like temperature, humidity, force, and monitor motion generated from the biological body. Then detected signal information is processed in another analyzing unit, following corresponding measures. The process has the potential to be simplified in an integrated unit, achieving diagnose and therapy at the same time. Gallium has been introduced and investigated as a vascular contrast agent [126], which provides a nonsurgical method for revolutionizing the current radiological imaging. Moreover, liquid metal has been utilized for tumor ablation taking advantage of its unique photothermal effect [127]. A recent micro-device integrated with actuation and sensing function was made for cardiac ablation [122], in which liquid metal helped to keep the catheter soft and flexible. These sensing and therapeutic combination attempts provide a new direction to multi-functional biosensors.

The liquid metal usually serves as a soft circuit in electrode, interconnection, and antennas. It enables highly sensitive signal detection and stable performance during deformation, which is almost the optimal material for electrically conductive biosensor. Meanwhile. the more physical and chemical features of liquid metal are under investigation for further exploring the applications. Other properties like electromagnetic, optical UV plasma [109], and photothermal effect [127] are also expected to be applied in biosensors. For example, gallium oxide’s selectivity and sensitivity enable its use as a gas-sensing element [22] through reactions between the metal oxide surface, chemisorbed oxygen, and gas molecules oxidizing and reducing gas. Moreover, liquid metal nanoparticles own unique UV plasma effects [109], opening a new way for optical sensors.

The soft electrode is the primary form of liquid metal in resistant and capacitive sensors. Moreover, there is ongoing research for more forms of liquid metal, for example, liquid metal particles are showing promise, possessing several notable attributes [128,129]. They are easily fabricated and have a unique metal-oxidation structure and the deformable ability to merge, self-healed, and transform, which enables their useful application in composites, soft electronics, and microfluidic, and so on. Firstly, liquid metal particles in soft composites can be engineered to possess excellent thermal and electrical conductivity by applying an external force to the elastomer and stimulating the rupture of oxidation film outside the liquid metal particles. Such composites are capable of serving as soft sensors for damage detection [51] or motion monitoring [130]. For electrical conductance, Ren [131] proposed a new conceptual droplet circuit and evaluated the probability of the droplets enabled functional circuits. Also, some sequent applications in capacitor [132], transistor [133], and free-standing microelectrodes [113] verified the feasibility of liquid metal microdroplets. While the tune of particle-particle contacts or adhesion is still a vitally important issue to be tackled, more potential biomedical use requires the construction of a computing chip or devices.

## 6. Conclusions

To summarize, the article systematically reviews the properties and typical applications in biosensors. Gallium-/bismuth-based liquid metals possess a good combination of fluidic and metallic properties. The low viscosity owing to its fluidic property enables liquid metal to be easily-fabricated, deformable, and controllable, opening up the possibility of new applications in diverse areas. The electrically conductive capability ascribable to the metallic character ensures a biosensor with liquid metal electrodes that are more precise and sensitive.

Besides, the intrinsic chemical reactivity of gallium or its intermediate oxide opens up more opportunities for liquid metals. In particular, the conformality and flexibility make liquid metal a better choice in wearable biosensors. Likewise, implantable biosensors are also possible in practice, attributed to the biocompatibility of liquid metal. Attributed to all these unconventional characteristics, liquid metals are capable of serving as a catalyst, electrodes, transistor, energy harvest in biosensors and be an alternative to the rigid structure in medical devices.

Finding optimal materials and more appropriate fabricating methods and making full use of diverse forms of liquid metal are vitally important for fully exerting its potential in biosensors.

Liquid metal has been found to serve as a facilitator in biosensors for detecting biological physical signals—including auditory sense, visual sense, tactile sense, gustation—and monitoring biochemistry changes like blood glucose. To extend the future clinical practices of new biomaterials, tremendous efforts are required to better understand their instinct properties and long-term performances. More biosensors based on liquid metal are expected to integrate detecting, monitoring, and therapeutic functions.

## Figures and Tables

**Table 1:** Summary of mechanical sensor based on liquid metal.

Types	Description	Fabrication Method	Sensitivity	Performance	Application	Ref
Pressure sensors	3D LM microfluidic channels incorporated inside the hydrogel matrix	3D casting molding; LM injection	1, 10, and 100 kPa	High modulus of the hydrogel; Poor sensitivity	Body-worn motion detector	[72]
	2D LM circuit: liquid-based thin PET film microfluidic tactile sensor	Lithography; LM injection	0.05 kPa^–1^	Range of 4 to 100 kPa	Wearable pressure sensor for real-time object grasping monitoring	[71]
	Triple-state liquid-based PET film microfluidic tactile sensor	Lithography; LM injection	(2–20) × 10^–3^ kPa^–1^	Range of 2 to 400 kPa	Severe mechanical load	[73]
	Based on GaIn microchannels and embedded equivalent Wheatstone bridge circuit	Lithography; LM injection	0.0835 kPa^−1^	Detection limit below 100 Pa with sub-50 Pa resolution	Detect and monitor heart rate from the wrist pulse	[74]
	3D-printed rigid microbump-integrated liquid metal-based soft pressure sensor	3D-printed; injection	0.158 kPa^−1^	Range of 0 to 80 kPa; without obvious hysteresis	Epidermal pulse and wireless wearable heel pressure monitoring system	[23]
	Liquid metal elastomer foam	Curing and dissolving	Capacitance: 0.992 pF kPa^−1^	Negative piezopermittivity; elastic modulus 7.8 kPa	Tactile sensor powered wirelessly	[75]
	Soft pressure microsensor with LM electrode	Lithography; LM injection	Resolution: 7.5 mmHg	Pressure range of 20–300 mmHg.	Measure blood pressure in vivo	[76]
	GaIn-BiInSn leakage-free electrodes-based sensors	Lithography; LM injection	0–0.45 Mpa^−1^	Double-capacitor sensor; leakage-free electrodes	-	[77]
Strain sensor	LM fiber sensor with hollow elastomeric capillaries	Injection	ΔC/L: 0 to 2.5%; Gauge factor: 0.66 to 0.82	Strain at 0–100%	Torsion and touch sensor	[64]
	LM fiber sensor with SEBS hollow fiber	Injection	ΔR/R: 0 to 50% (nonlinear)	Strain at 0–700%	Combinations of good conductivity and stretchability for conductive fibers	[53]
	LM fiber sensor with PDMS hollow fiber	Injection	ΔR/R: 0 to 100%; Gauge factor: 2.2 to 3.4	Strain at 0–140%	Low detection limit (0.3% strain); good repeatability	[62]
	Microchannel in commercially available resin	Stereolithography-based 3D printing	ΔR/R: −2.5 to 2.5%	-	Minimum resolution angle of 1° (70° to −70°)	[95]
	Surface-embedded metal in polymeric hosts	Casting and peeling off steps	ΔR/R: 0 to 60%(nonlinear)	Strain at 0 to 500%; stress (~0.4 MPa)	Repeatability (ΔR/R <3%)	[96]
	Hydrogel shells and LM droplets	Polymerization colloidal cross-linker	ΔR/R:0 to 700%; gauge factor: 1.54	Toughness (3.54 MJ/m^3^); fracture stress (1.26 MPa)	Self-healing strain sensor	[98]

**Table 2:** Summary of gas sensor based on liquid metal.

Type	Detection Gas	Detection Sensitivity	Working Temperature	Evaluation	Ref
β-Ga_2_O_3_ film with Au dispersion	CO	4–100 ppm	>550 °C	Response in seconds (>700 °C)	[99]
β-Ga_2_O_3_ film (chemisorption)	H_2_	10^−3^ bar	400–650 °C	Response in 10 s; high working temperature	[100]
Monoclinic Ga_2_O_3_ crystal structure (Ga with In, Sn, and Zn)	NO_2_ and H_2_	4.5 ppm (NO_2_) 1.0% (H_2_)	150 °C (NO_2_) 350 °C (H_2_)	Only discuss the temperature response	[22]
Ga_2_O_3_-WO_3_ heterostructures	C_2_H_5_OH	1 to 600 ppm	275 °C	Response in hundred seconds; ultrathin (10 nm)	[101]
β-Ga2O3 (physisorption)	NO_2_ and NH_3_	1–12 ppm (NO_2_) 20–99 ppm (NH_3_)	100 °C	Poor durability	[102]

**Table 3:** Summary of temperature sensors based on liquid metal.

Types	Fabrication Method	Detection Sensitivity	Detection Range	Evaluation	Ref
Ga and matching metal wires	Direct printing	0.5 °C	0 to 200 °C	High precision; Tiny size	[3]
Ga-Bi alloy micro-thermocouple	Microfluidic injection	−10.54 μV/K	25 to 83 °C	Stable under 90° bending	[103]
EGaInSn and SWCNT	Multilayers assemble	1.0% °C^−1^	15 to 45 °C	Highly stretchable	[106]
